# Hymecromone: a clinical prescription hyaluronan inhibitor for efficiently blocking COVID-19 progression

**DOI:** 10.1038/s41392-022-00952-w

**Published:** 2022-03-18

**Authors:** Shuai Yang, Yun Ling, Fang Zhao, Wei Li, Zhigang Song, Lu Wang, Qiuting Li, Mengxing Liu, Ying Tong, Lu Chen, Daoping Ru, Tongsheng Zhang, Kaicheng Zhou, Baolong Zhang, Peng Xu, Zhicong Yang, Wenxuan Li, Yuanlin Song, Jianqing Xu, Tongyu Zhu, Fei Shan, Wenqiang Yu, Hongzhou Lu

**Affiliations:** 1 Laboratory of RNA Epigenetics, Institutes of Biomedical Sciences & Shanghai Public Health Clinical Center & Department of General Surgery, Huashan Hospital, Cancer Metastasis Institute Shanghai Medical College, Fudan University, Shanghai, P. R. China; 2 Shanghai Public Health Clinical Center, Fudan University, Shanghai, P. R. China; 3 Department of Radiology, Shanghai Public Health Clinical Center, Fudan University, Shanghai, P. R. China; 4 Zhongshan Hospital, Fudan University, Shanghai, P. R. China; 5 The Third People’s Hospital of Shenzhen, Shenzhen, P. R. China

**Keywords:** Infectious diseases, Translational research

## Abstract

Currently, there is no effective drugs for treating clinically COVID-19 except dexamethasone. We previously revealed that human identical sequences of SARS-CoV-2 promote the COVID-19 progression by upregulating hyaluronic acid (HA). As the inhibitor of HA synthesis, hymecromone is an approved prescription drug used for treating biliary spasm. Here, we aimed to investigate the relation between HA and COVID-19, and evaluate the therapeutic effects of hymecromone on COVID-19. Firstly, HA was closely relevant to clinical parameters, including lymphocytes (*n* = 158; *r* = −0.50; *P* < 0.0001), C-reactive protein (*n* = 156; *r* = 0.55; *P* < 0.0001), D-dimer (*n* = 154; *r* = 0.38; *P* < 0.0001), and fibrinogen (*n* = 152; *r* = 0.37; *P* < 0.0001), as well as the mass (*n* = 78; *r* = 0.43; *P* < 0.0001) and volume (*n* = 78; *r* = 0.41; *P* = 0.0002) of ground-glass opacity, the mass (*n* = 78; *r* = 0.48; *P* < 0.0001) and volume (*n* = 78; *r* = 0.47; *P* < 0.0001) of consolidation in patient with low level of hyaluronan (HA < 48.43 ng/mL). Furthermore, hyaluronan could directly cause mouse pulmonary lesions. Besides, hymecromone remarkably reduced HA via downregulating *HAS2/HAS3* expression. Moreover, 89% patients with hymecromone treatment had pulmonary lesion absorption while only 42% patients in control group had pulmonary lesion absorption (*P* < 0.0001). In addition, lymphocytes recovered more quickly in hymecromone-treated patients (*n* = 8) than control group (*n* = 5) (*P* < 0.05). These findings suggest that hymecromone is a promising drug for COVID-19 and deserves our further efforts to determine its effect in a larger cohort.

## Introduction

As of February 22, 2022, more than 424 million infections of COVID-19 have been confirmed worldwide, resulting in 5,890,312 deaths according to the WHO Coronavirus Disease (COVID-19) Dashboard. Significantly, the emergence of B.1.617.2 (Delta) variant has caused breakthrough infections and exacerbated this epidemic,^1^ which highlights the importance of exploring the common strategy for the treatment of COVID-19 caused by diverse SARS-CoV-2 variants. With such a rapid variation and high mortality of SARS-CoV-2, there is an urgent need for appropriate treatment to prevent the deterioration of moderate and severe COVID-19, and to reduce the mortality.

Generally, there are two approaches to fight against virus-related diseases. As for COVID-19, one approach is to target SARS-CoV-2 virus itself to prevent the replication or entry to the human cell. For example, as one of the ribonucleoside prodrugs, molnupiravir could destroy the viral genomic replication and ultimately inhibit its replication.^2^ Another small-molecule drug, PF-07321332, disturbs the replication of SARS-CoV-2 through inhibiting the 3CL protease.^3^ Besides, neutralizing antibodies function as goalkeepers to restrain the SARS-CoV-2 entry into cells via binding the surface antigens,^4^ whose therapeutic effect may be decreased due to the rapid mutation of SARS-CoV-2. Another approach against COVID-19 should be to block the interaction between SARS-CoV-2 and humans, which could be an effective strategy to overcome the continuous mutation of SARS-CoV-2. When it comes to this aspect, it is vital to find the key pathogenic factors of COVID-19 by decoding their underlining pathogenic mechanism.

Currently, almost all the progress to developing drugs for COVID-19 is to focus on the infection and replication of SARS-CoV-2.^5–7^ Instead, very few drugs target the pathogenic factors of COVID-19, which may be due to the lack of specific targets. Recently, we identified five identical sequences between the genomes of SARS-CoV-2 and human, termed Human Identical Sequences (HIS), which can promote the accumulation of hyaluronan by activating hyaluronic acid synthase 2 (HAS2).^8^ Accordingly, adult respiratory distress syndrome (ARDS) is one of the typical clinical symptoms in severe COVID-19 patients,^9^ and hyaluronic acid (HA) is accumulated in the lung of patients with ARDS.^10^ Of note, hyaluronan is higher in the lung tissue of deceased COVID-19 patients than that in healthy people.^11^ Hyaluronan regulates diverse biological and pathological processes involved in inflammation responses, immune responses, and tissue injury.^12^ Meanwhile, the other common features of severe COVID-19 patients include inflammatory cytokine storm, lymphocytopenia, and ground-glass opacity (GGO) in the lung.^13,14^ These evidence raise the possibility that hyaluronan induced by HIS may be connected with the clinical symptoms of COVID-19 and serve as an optional therapeutic target. Fortunately, as the inhibitor of HA synthesis, hymecromone is an approved prescription oral drug used for treating biliary spasms in Europe and Asia.^15^ Accordingly, it deserves our time to confirm whether hymecromone could be repurposed to as a therapeutic drug for COVID-19 after we identify the potential roles of hyaluronan in COVID-19.

Here, we found that hyaluronan was markedly increased in patients with pulmonary lesions and significantly correlated to the clinical parameters for the prediction of COVID-19 severity, including lymphocytes, C-reactive protein (CRP), D-dimer, and fibrinogen. Furthermore, hyaluronan directly induced GGO and the consolidation of the lung in mice, similar to the characteristics of computed tomographic (CT) scans in COVID-19 patients with pulmonary lesions. Excitingly, inhibition of hyaluronan production with hymecromone could significantly improve clinical manifestations involved in lymphopenia and pulmonary lesion in COVID-19 patients. Repurposing hymecromone as a drug for COVID-19 could be a hopeful strategy to overcome SARS-CoV-2 once its therapeutical effect is confirmed in a larger cohort in the future.

## Results

### Hyaluronan is a considerable biomarker to predict COVID-19 progression

**Fig. 1:**
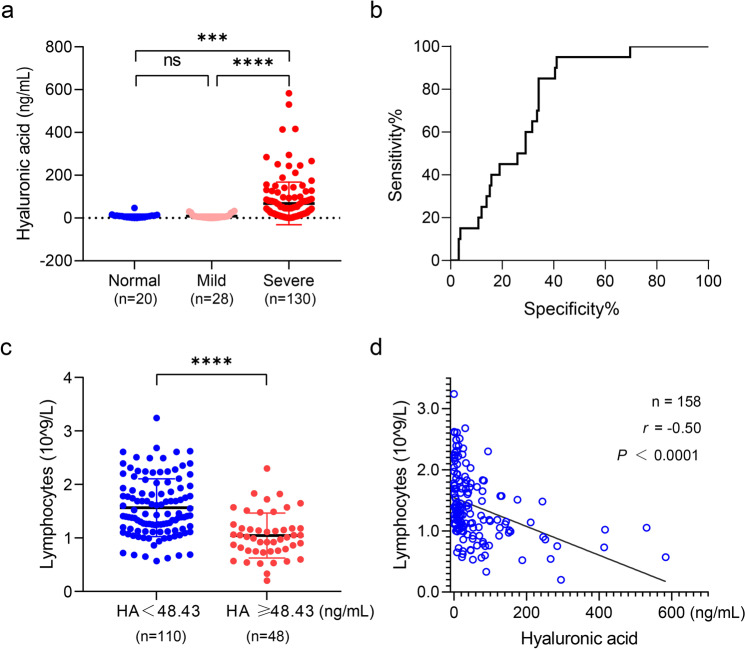
The increase of hyaluronan is related to the severity in COVID-19 patients. **a** The plasma hyaluronan level of COVID-19 patients and normal healthy subjects were evaluated by ELISA. The classification of mild and severe of COVID-19 was based on pulmonary lesions of chest CT. **b** ROC of the plasma hyaluronan level in normal volunteers and COVID-19 patients. The 48.43 ng/mL of hyaluronan is the cutoff value to distinguish the normal subjects and COVID-19 patients. **c** Counts of lymphocytes upon admission plotted against hyaluronic acid. **d** Two-tailed Spearman’s correlation analysis was performed to evaluate the correlation between hyaluronan and lymphocyte counts. Data are presented by mean ± SD. The significant difference in (**a**) and (**c**) was analyzed by the Mann–Whitney test. *P < 0.05; **P < 0.01; ***P < 0.001; ****P < 0.0001; ns, not significant

A total of 158 COVID-19 patients in Shanghai Public Health Clinical Center (SPHCC) were conducted to investigate the potential relationship between hyaluronan and the typical clinical indicators for COVID-19. Specifically, 18% (28 of 158) patients without pulmonary lesions were mild, while the other patients (130 of 158) with pulmonary lesions including GGO and consolidation were severe. The plasma HA level showed no significant difference between healthy subjects and mild COVID-19 patients, while the plasma HA level in severe COVID-19 patients was significantly higher than that in the other two groups (Fig. 1a), indicating there is a potential relation between hyaluronan and pulmonary lesions. Further receiver operating characteristic curve (ROC) analysis identified that the 48.43 ng/mL of hyaluronan was sensitive for distinguishing COVID-19 patients and healthy subjects (Fig. 1b). Then, 158 COVID-19 patients were divided into low HA group (*n* = 110, HA < 48.43 ng/mL) and high HA group (*n* = 48, HA ≥ 48.43 ng/mL). The general characteristics of these patients were shown in Table 1. Specifically, nearly all patients with high HA levels had pulmonary lesions based on CT scans.

**Table 1:** The clinical features of the 158 patients in the observational study

	HA < 48.43 ng/mL	HA ≥ 48.43 ng/mL
Numbers	110	48
Age		
Median (range)—year	36.5 (19–75)	53.5 (23–84)
≤29, *n* (%)	23 (20.91%)	6 (12.50%)
30–39, *n* (%)	43 (39.09%)	6 (12.50%)
40–49, *n* (%)	22 (20.00%)	5 (10.42%)
50–59, *n* (%)	4 (3.64%)	16 (33.33%)
≥60, *n* (%)	18 (16.36%)	15 (31.25%)
Gender		
Female (%)	37 (33.64%)	20 (41.67%)
Male (%)	73 (66.36%)	28 (28.33%)
Disease severity^a^		
Mild, *n* (%)	27 (24.55%)	1 (2.08%)
Severe, *n* (%)	83 (75.45%)	47 (97.92%)
Comorbidities		
None, *n* (%)	72 (65.45%)	30 (62.50%)
Diabetes^b^, *n* (%)	4 (3.64%)	5 (10.42%)
Hypertension^c^, *n* (%)	13 (11.82%)	9 (18.75%)
Steatohepatitis^d^, *n* (%)	1 (0.91%)	1 (2.08%)
Asthma^e^, *n* (%)	1 (0.91%)	0 (0.00%)
Rhinitis^f^, *n* (%)	10 (9.09%)	0 (0.00%)
Hepatitis^g^, *n* (%)	3 (2.73%)	1 (2.08%)
Blood disease^h^, *n* (%)	1 (0.91%)	2 (4.17%)

^a^Disease severity was diagnosed at the patient’s admission

^b^Patients with diabetes were treated with one or more drugs as follows: Metformin, Glimepiride, and Glipizide

^c^Patients with hypertension were treated with one or more drugs as follows: Amlodipine, Amlodipine, Losartan, Levamlodipine, Felodipine, Benazepril, and Irbesartan

^d, e, f, g, h^There was no documented history for the drug treating steatohepatitis, hepatitis, and blood disease including hyperuricemia, hyperlipemia, and anemia

Lymphopenia is one of the typical clinical symptoms in severe COVID-19 patients.^16^ It is reported that hyaluronan can result in the death of the activated T cell.^17^ Here, we found that lymphocytes were markedly decreased in the high HA group of COVID-19 patients (Fig. 1c). As shown in Fig. 1d, hyaluronan was negatively correlated with lymphocytes (*n* = 158; *r* = −0.50; *P* < 0.0001). The subsets of T lymphocytes, CD4^+^ T cells, CD8^+^ T cells, and CD45^+^ T cells were also significantly reduced in COVID-19 patients with high HA (Supplementary Fig. 1a–c). Similarly, hyaluronan was negatively correlated with the CD4^+^ T cells (*n* = 151; *r* = −0.44; *P* < 0.0001), CD8^+^ T cells (*n* = 151; *r* = −0.53; *P* < 0.0001), and CD45^+^ T cells (*n* = 151; *r* = −0.49; *P* < 0.0001) (Supplementary Fig. 1d–f).

**Fig. 2:**
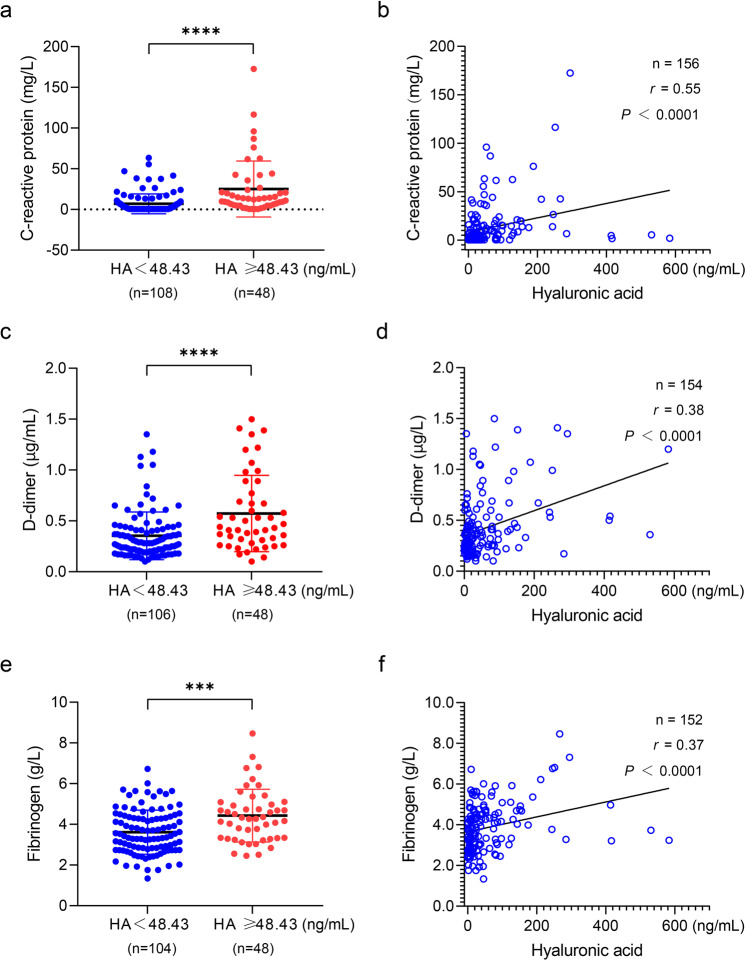
The plasma level of hyaluronan as a typical indicator of COVID-19 patients in the clinical setting. **a** C-reactive protein of COVID-19 patients is showing based on the threshold of 48.43 ng/mL of hyaluronan. **b** The relation of C-reactive protein against hyaluronic acid was determined by two-tailed Spearman’s correlation analysis. **c** D-dimer of COVID-19 patients is showing based on the threshold of 48.43 ng/mL of hyaluronan. **d** The relation of D-dimer against hyaluronic acid was determined by two-tailed Spearman’s correlation analysis. **e** Fibrinogen of COVID-19 patients are showing based on the threshold of 48.43 ng/mL of hyaluronan. **f** The relation of fibrinogen against hyaluronic acid was determined by two-tailed Spearman’s correlation analysis. Data in (a, c, and e) are presented by mean ± SD. The significant difference was confirmed by the Mann–Whitney test. *P < 0.05; **P < 0.01; ***P < 0.001; ****P < 0.0001; ns, not significant

Inflammation is another common clinical symptom involved in COVID-19 patients, usually assessed via CRP. Noteworthily, the low molecular weight of hyaluronan is an important inflammation mediator.^12^ Surprisingly, we found that CRP markedly increased in the high HA group (Fig. 2a) and was positively correlated with hyaluronan (*n* = 156; *r* = 0.55; *P* < 0.0001) (Fig. 2b). Recent studies revealed abnormal blood coagulation in COVID-19 patients.^13^ Clinically, D-dimer and fibrinogen are commonly used to assess a patient’s blood coagulation. We found that both of them were higher in COVID-19 patients with high HA and were positively correlated with hyaluronan (Fig. 2c–f).

Collectively, these results reveal that the increase of hyaluronan is significantly relevant to the reduction of lymphocytes and the upregulation of CRP, D-dimer, and fibrinogen in COVID-19 patients, suggesting hyaluronan may play a key role during the clinical progression of COVID-19.

### Hyaluronan is fundamental for ground-glass opacity formation in the lung of COVID-19 patients

**Fig. 3:**
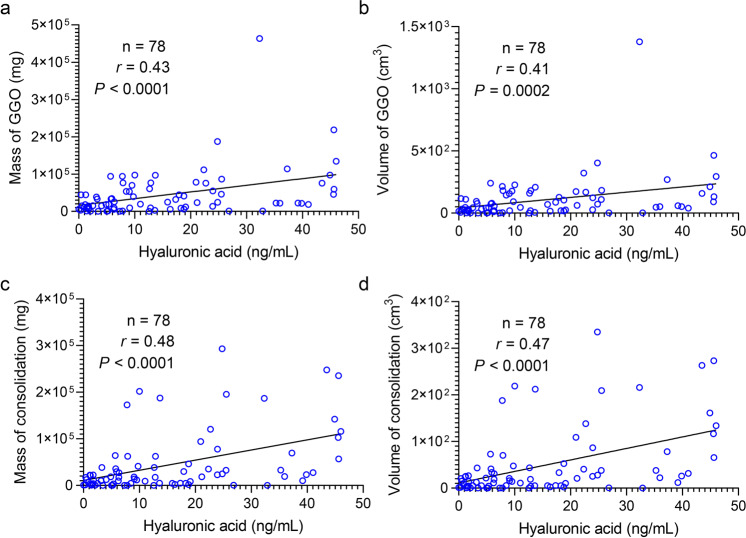
Correlation between hyaluronan and pulmonary lesions in COVID-19 patients with a low level of hyaluronan (HA < 48.43 ng/mL). **a**, **b** Scatter plot showing relation on the mass (**a**) and volume (**b**) of GGO against hyaluronic acid. **c**, **d** Scatter plot showing relation on the mass (**c**) and volume (**d**) of the consolidation region against hyaluronic acid. Mass and volume of pulmonary lesions were calculated via the automatic lung segmentation technology of AI based on CT images of severe COVID-19 patients. GGO was defined a range from −750 HU to −300 HU, and the consolidation region was defined from −300 HU to 50 HU. Two-tailed Spearman’s correlation analysis was performed to identify the relation of pulmonary lesions against hyaluronic acid

It is well-known that ground-glass opacity of lungs is the other typical clinical manifestation of COVID-19 patients,^18^ which can develop into consolidation. We quantified the mass and volume of the lung lesion regions involved in GGO and consolidation in 120 COVID-19 patients using uAI-Discover-NCP (beta version). Here, we classified these patients into two groups based on the hyaluronan level. In general, GGO was defined in a range from −750 HU to −300 HU, and the consolidation region was defined from −300 HU to 50 HU.^19^ Notably, hyaluronan was positively correlated with the mass (*n* = 78; *r* = 0.43; *P* < 0.0001) and volume (*n* = 78; *r* = 0.41; *P* = 0.0002) of GGO in patients with low level of hyaluronan (HA < 48.43 ng/mL), which was also the case in the mass (*n* = 78; *r* = 0.48; *P* < 0.0001) and volume (*n* = 78; *r* = 0.47; *P* < 0.0001) of consolidation (Fig. 3). However, there was no significant correlation between hyaluronan and pulmonary lesions including GGO and consolidation in patients with a high level of hyaluronan (HA ≥ 48.43 ng/mL) (Supplementary Fig. 2). These findings suggested that hyaluronan could be related to the initial formation of GGO and consolidation. Recent research found that there is jelly-like liquid in the lung of COVID-19 patients.^9^ Given that hyaluronan can absorb water reaching 1000 times its molecular weight,^20^ we hypothesize that hyaluronan may be one of the determinants for GGO of the lung in COVID-19 patients.

**Fig. 4:**
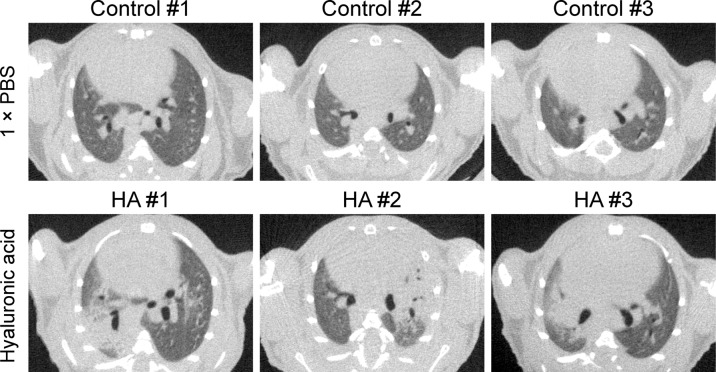
Hyaluronan directly induces GGO and consolidation in adult mice. Represented CT images of lungs in mice with different treatments are shown. Adult C57BL/6 mice were used to assess whether hyaluronic acid induces pulmonary lesions. We directly delivered hyaluronan (200–400 kDa) to the trachea (*n* = 3), and 1× PBS treatment was as the control group (*n* = 3). Then, we monitored the lungs of mice in two groups via QuantumGX microCT at the fourth day

To confirm our hypothesis, we delivered hyaluronan intratracheally to the lungs in male mice. Significantly, CT images showed that GGO and consolidation of lung occurred in mice treated with hyaluronan (*n* = 3) while there were no pulmonary lesions in mice treated with 1× PBS (*n* = 3) (Fig. 4). Therefore, these findings supported that hyaluronan acts as a critical material for the formation of GGO and consolidation of the lung in COVID-19 patients, indicating that inhibition of HA synthesis may be a promising strategy for relieving pulmonary lesions in COVID-19 patients.

### Hymecromone significantly decreases hyaluronan by downregulating *HAS2/HA3*

**Fig. 5:**
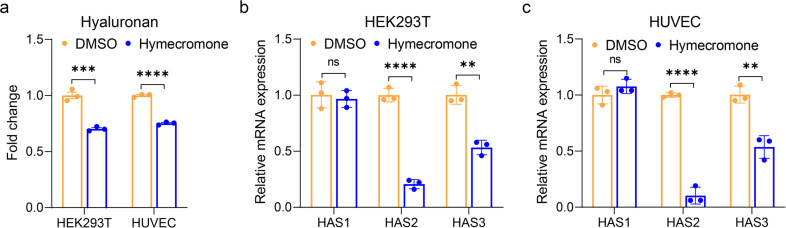
Hymecromone decreases hyaluronan by downregulating hyaluronic acid synthases. **a** ELISA detected the hyaluronic acid level of culture medium in HEK293T and HUVEC treated with DMSO or hymecromone (200 μg/mL). The fold change of hyaluronic acid was normalized to DMSO. **b** RT-qPCR evaluated the mRNA levels of *HAS1*, *HAS2*, and *HAS3* in HEK293T treated with DMSO or hymecromone (200 μg/mL). **c** RT-qPCR evaluated the mRNA levels of *HAS1*, *HAS2*, and *HAS3* in HUVEC treated with DMSO or hymecromone (200 μg/mL). All experiments were repeated independently in triplicate. Data are presented by mean ± SD. The significant difference was confirmed by unpaired *t* test. *P < 0.05; **P < 0.01; ***P < 0.001; ****P < 0.0001; ns, not significant

Based on these findings, we realized that reduction of HA production could be an alternative therapeutic strategy for COVID-19, especially for patients with pulmonary lesions. As a derivative of coumarin, 4-MU was shown to inhibit the production of HA.^21^ Fortunately, we noticed there was a commercial drug-containing 4-MU, hymecromone, which is an approved prescription drug in China, USA and Europe, even acting as an over-the-counter drug in some areas. We first verified the inhibitory effect of hymecromone on HA production in HEK293T and HUVEC cells. As expected, HA from culture medium in HEK293T and HUVEC cells treated with hymecromone (200 μg/mL) were significantly lower than that in DMSO-treated cells (Fig. 5a). There are three known hyaluronic acid synthases, including HAS1, HAS2, and HAS3 (HAS1/2/3). As shown in Fig. 5b, c, hymecromone treatment remarkably downregulated the expression of *HAS2/HAS3*, but did not affect the expression of *HAS1* in HEK293T and HUVEC cells. Therefore, hymecromone inhibits the production of hyaluronan by decreasing *HAS2/HAS3* expression.

### Hymecromone accelerates the recovery of clinical manifestations in COVID-19 patients

**Fig. 6:**
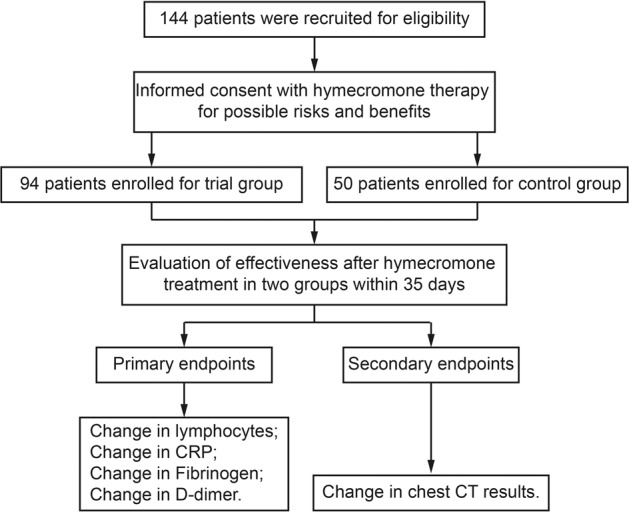
The Consort Diagram of recruited COVID-19 patients for this clinical trial. Among these patients, 94 patients were in the trial group while 50 patients were in the control group. Changes in lymphocytes, CRP, fibrinogen, and D-dimer were set as the primary endpoints and change in chest CT results was set as the secondary endpoint

To further assess whether hymecromone is efficient in improving the clinical parameters of COVID-19, we recruited 144 confirmed COVID-19 patients (Table 2). Among these patients, 94 (65%) patients with hymecromone treatment were in the clinical trial group, while 50 (35%) patients with support treatment were in the control group (Fig. 6). Given the significant correlation between hyaluronan and clinical indicators, we set changes in lymphocytes, CRP, fibrinogen, and D-dimer as the primary endpoints, and the changes in chest CT results as the secondary endpoint. To objectively evaluate the effect of hymecromone on COVID-19, we selected COVID-19 patients with abnormal clinical indicators in these two groups. Specifically, there were five patients with decreased lymphocytes, four patients with increased CRP, seven patients with increased fibrinogen, and eight patients with increased D-dimer in the control group. In the clinical trial group, there were eight patients with decreased lymphocytes, five patients with increased CRP, seven patients with increased fibrinogen, and six patients with increased D-dimer (Supplementary Table 1).

**Table 2:** The clinical features of enrolled patients and subgroups

	Control group	Experimental group
Administration		
Conventional treatment	√	√
Hymecromone treatment	×	√
Numbers	50	94
Age		
Median (range)—year	35 (21–69)	41.5 (21–77)
≤29, *n* (%)	13 (26.00%)	21 (22.34%)
30–39, *n* (%)	16 (32.00%)	19 (24.47%)
40–49, *n* (%)	10 (20.00%)	23 (24.47%)
50–59, *n* (%)	6 (12.00%)	22 (23.40%)
≥60, *n* (%)	5 (10.00%)	5 (5.32%)
Gender		
Female (%)	14 (28.00%)	31 (32.98%)
Male (%)	36 (72%)	63 (67.02%)
Disease severity^a^		
Mild, *n* (%)	21 (42.00%)	28 (29.79%)
Severe, *n* (%)	29 (58.00%)	66 (70.21%)
Comorbidities		
None, *n* (%)	36 (72.00%)	76 (80.85%)
Diabetes^b^, *n* (%)	5 (10.00%)	6 (6.38%)
Hypertension^c^, *n* (%)	5 (10.00%)	9 (9.57%)
Steatohepatitis^d^, *n* (%)	3 (6.00%)	3 (3.19%)
Asthma^e^, *n* (%)	0 (0.00%)	1 (1.06%)
Hypohepatia^f^, *n* (%)	0 (0.00%)	1 (1.06%)
Hepatitis^g^, *n* (%)	2 (4.00%)	3 (3.19%)
Blood diseases^h^, *n* (%)	1 (2.00%)	1 (1.06%)

^a^Disease severity was diagnosed at the patient’s admission

^b^Patients with diabetes were treated with one or more drugs as follows: Metformin, Glimepiride, Jardiance, and Sitagliptin

^c^Patients with hypertension were treated with one or more drugs as follows: Nifedipine, Perindopril, Amlodipine, Telmisartan, Felodipine, and Irbesartan

^d^Patients with steatohepatitis were treated with Dangfei Liganning capsules

^e^Patients with asthma were treated with Salmeterol

^f^There was no documented history for the drug treating the hypohepatia

^g^Patients with hepatitis were treated with Tenofovir

^h^Blood diseases included hyperuricemia, hyperlipemia, and anemia. There was no documented history for the drug treating these blood diseases

**Fig. 7:**
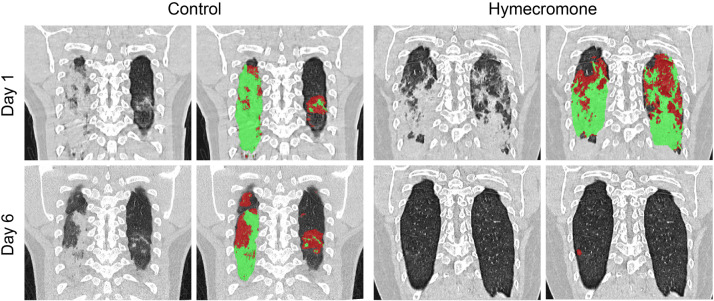
Represented CT images of COVID-19 patients with support or hymecromone treatment. The original CT results are shown in black-and-white images of two groups while the marked lesion regions of CT results are shown in color images of two groups. Red regions indicate GGO, and green regions indicate consolidation

First, we focused on the change of pulmonary lesions in COVID-19 patients during hymecromone treatment. We determined the situation of pulmonary lesions, including GGO and consolidation based on CT quantitative analysis. Given that there were few patients with pulmonary lesions in the control group compared to the hymecromone-treated group, we added the patients with pulmonary lesions based on the CT scans from the 158 patients into the control group. Surprisingly, all the patients with hospitalization days <14 days had improved pulmonary lesions after hymecromone treatment (Table 3). The percentage of pulmonary lesion improvement in patients with hymecromone treatment was significantly higher than that in the control group (*P* = 0.0002) when the hospitalization days were between 14 and 28 days. For the patients with hospitalization between 28 and 35 days, the percentage of pulmonary lesion improvement rate was 86% (6 of 7) patients in the trial group, and 45% (2 of 9) in the control group, respectively. In total, 89% (41 of 46) patients with hymecromone treatment had pulmonary lesion absorption, while only 42% (42 of 100) in the control group had pulmonary lesion absorption (*P* < 0.0001). Different regions of pulmonary lesions in typical cases in two groups were gradually absorbed (Fig. 7). As such, patients have better improvement of pulmonary lesions after hymecromone treatment. Thus, hymecromone could promote the pulmonary lesion absorption of COVID-19.

**Table 3:** Comparison of the effect of hymecromone on pulmonary lesions in COVID-19 patients classified by different hospitalization

Hospital stay^a^	CT change	Control group	Experimental group	*P* value^*^
X < 14	Improvement	10 (42%)	11 (100%)	Yes
	Exacerbation	14 (58%)	0 (0%)	
14 ≤ X ≤ 28	Improvement	30 (45%)	24 (86%)	*P* = 0.0002
	Exacerbation	37 (55%)	4 (14%)	
28 ≤ X ≤ 35	Improvement	2 (22%)	6 (86%)	*P* = 0.0406
	Exacerbation	7 (78%)	1 (14%)	
Total numbers	Improvement	42 (42%)	41 (89%)	*P* < 0.0001
	Exacerbation	58 (58%)	5 (11%)	

^a^The number of days was used to classify the hospital day

*P value was calculated by Fisher’s exact test using GraphPad Prism

**Fig. 8:**
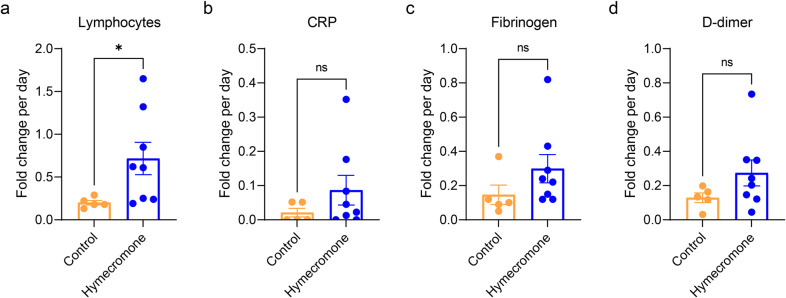
Hymecromone accelerate the recovery of COVID-19 patients from lymphocytopenia. Changes in lymphocytes (**a**), CRP (**b**), fibrinogen (**c**), and D-dimer (**d**) were calculated as the fold change of diverse clinical indicators per day in patients with lymphocytopenia. Data are presented by mean ± SEM. The significant difference was confirmed by the Mann–Whitney test. *P < 0.05; **P < 0.01; ***P < 0.001; ****P < 0.0001; ns, not significant

Then, we calculated and compared the fold changes of these various clinical indicators per day in these two groups with lymphopenia, CRP elevation, fibrinogen elevation, and D-dimer elevation, respectively. First, we reanalyzed the incidence of lymphopenia, and the elevation of CRP, fibrinogen, and D-dimer in these two groups using Fisher’s exact test (Supplementary Table 1). There is no statistical difference of these events between the control and hymecromone-treated groups. As shown in Fig. 8, the fold change of lymphocytes per day was remarkably higher in hymecromone-treated patients, compared to the control group. Similarly, the fold changes of CRP or fibrinogen in hymecromone-treated patients were higher than that in the control group (Supplementary Figs. 3–4). These results implied that hymecromone contributes to the improvement of clinical parameters of COVID-19. Moreover, the fold change of D-dimer tended to be improved, but it is not significant between the control group and clinical trial group (Supplementary Fig. 5), which may be due to the limited number of patients. Besides, there are no adverse reactions observed in this clinical trial. Specially, there is no significant difference of the biochemical indicators to assess the functions of the liver (such as alanine aminotransferase (ALT), aspartic transaminase (AST), and alkaline phosphatase (ALP) and kidney (such as urea, and creatinine) between control and hymecromone-treated groups (Supplementary Fig. 6), indicating that the current administration of hymecromone did not injury the functions of the liver and kidney in COVID-19 patients.

These findings demonstrated that hymecromone is a promising drug for effectively improving the clinical manifestations of COVID-19.

## Discussion

Our previous study has found that HIS of SARS-CoV-2 could promote hyaluronan upregulation by NamiRNA-Enhancer network during the progression of COVID-19,^8^ indicating that reduction in hyaluronan may be an alternative therapeutic strategy for COVID-19, which is supported by the other reports found that hyaluronan was increased in severe COVID-19 patients.^11,22,23^ As an inhibitor of HA synthesis, hymecromone is an approved drug for biliary spasms treatment. Here, we aimed to assess whether hymecromone could promote the prognosis of COVID-19.

Hyaluronan is an alternative biomarker to predict COVID-19 progression. We found that hyaluronan was significantly elevated in severe COVID-19 patients. Consistent with our results, increases in hyaluronan were also confirmed in severe or critical patients with COVID-19.^23^ Notably, hyaluronan is accumulated in the bronchoalveolar lavage fluid (BALF) and serum samples of patients with ARDS.^24^ We also found that hyaluronan was significantly correlated with lymphocytes, CRP, D-dimer, and fibrinogen. These clinical indicators are proven biomarkers for the clinical progression of COVID-19.^25^ All these findings demonstrated that hyaluronan was relevant to COVID-19 progression. In addition, we showed that hyaluronan positively correlates with quantified chest CT results, a recently identified parameter to predict the severity of COVID-19.^26^ This result further supported that hyaluronan can serve as a better biomarker for predicting the clinical progression of COVID-19.

Most importantly, our results provide vital insights into the typical clinical symptoms occurring in COVID-19. As we all know, GGO is one of the typical CT manifestations of COVID-19 patients.^27^ However, the pathophysiologic mechanism of GGO is still unclear. We showed that hyaluronan can cause GGO and consolidation of the lung in mice, providing the direct evidence that hyaluronan is fundamental for GGO formation. Consistent with our results, transcriptome sequencing of cells in BALF from COVID-19 patients revealed differentially altered genes enriched in the hyaluronan metabolic pathway.^22^ The appearance of GGO were further interpreted by the facts that the water absorption of hyaluronan can reach 1000 times its molecular weight,^20^ possibly resulting in the formation of jelly-like liquid in the lung of COVID-19 patients.^9^ In addition to GGO, lymphopenia is another typical clinical symptom in severe COVID-19 patients.^16^ We found that the crucial subsets of T lymphocytes, CD4^+^ T cells, CD8^+^ T cells, and CD45^+^ T cells were also significantly decreased in COVID-19 patients with high HA. Meanwhile, the negative correlation between hyaluronan and diverse T-cell subsets confirmed that hyaluronan was associated with the reduction of T lymphocytes. In line with this sight, hyaluronan can induce the death of the activated T cells by binding to its ligand CD44.^17^ Notably, CD4^+^ T lymphocytes become rapidly activated after SARS-CoV-2 infection,^28^ which provides the condition needed for the binding of HA to CD44 located in activated T cells, leading to their death. In other words, T-cell exhaustion mediated by hyaluronan may potentially underly lymphopenia of COVID-19 patients.

Hymecromone could accelerate the recovery of clinical manifestations in COVID-19 patients. At present, the therapeutic strategy for COVID-19 is mainly symptomatic supportive treatment in clinic.^29^ Here, we found that hymecromone inhibited hyaluronan production by suppressing the expression of *HAS2/3*. Additionally, hymecromone significantly improved lymphopenia of COVID-19 patients, indicating that declines in hyaluronan via hymecromone administration promote the recovery of lymphopenia. Likewise, hymecromone decreased the CRP and fibrinogen elevation of COVID-19 patients. Also remarkably, hymecromone accelerated pulmonary lesions absorption. It has already been reported that dexamethasone and metformin can significantly decrease the mortality of patients with severe COVID-19.^30–32^ Accordingly, these results may be explained by the reports that dexamethasone and metformin can also rapidly decline hyaluronan synthesis by downregulating *HAS2* expression,^33,34^ which may contribute the therapeutic effects of these two drugs on COVID-19. In addition, we found most HIS is quite conservative by analyzing 159258 genomes of SARS-CoV-2 (unpublished data), suggesting hyaluronan caused by HIS may be an important therapeutic target for diverse SARS-CoV-2 variants. All these evidence support that hymecromone could be a potential and effective drug for COVID-19 patients even infected with delta variant.

There are several limitations to our study. First, the total number of patients involved in our clinical trial is relatively insufficient, which requires a larger sample and multi-center clinical study in the future. Specially, the randomization procedure would be processed strictly following the randomized controlled trial rather than dependent on hospitalization number. Second, we did not evaluate whether hymecromone can reduce the mortality rates of COVID-19 patients because of no critical patients in this clinical trial. Third, given that the exact value of hyaluronan may be different using diverse methods, it is necessary to identify the hyaluronan concentration to clinically distinguish the healthy subjects and COVID-19 patients by uniform standard methods.

Overall, the increased hyaluronan is significantly correlated with the decreased lymphocytes and pulmonary lesions of COVID-19 patients. Hymecromone administration can markedly improve the clinical manifestations of COVID-19 patients. Thus, hymecromone could be a potential and efficient drug for COVID-19 therapy, which is needed to further clarify its effect in a larger sample of clinical trials. Our findings highlight that inhibiting the synthesis of hyaluronan with specific drugs is a promising therapeutic strategy for COVID-19.

## Materials and methods

### Patient enrollment and experiment animal

All COVID-19 patients enrolled in this study have received written informed consent upon admission into the SPHCC, the designated hospital for COVID-19 patients in Shanghai, China.^35^ This study was approved by the Ethics Committee of the SPHCC (YJ-2020-S123-02) and registered in the Chinese Clinical Trial Registry (ChiCTR2100051688). COVID-19 for patients was confirmed based on the Guidelines of the Diagnosis and Treatment of New Coronavirus Pneumonia (version 7) published by the National Health Commission of China. Exclusion criteria for COVID-19 patients are listed as below: (1) serious non-infectious pulmonary diseases, including pulmonary tumor, pulmonary edema, atelectasis, pulmonary embolism, pulmonary eosinophilic infiltration, pulmonary vasculitis etc.; (2) severe liver and kidney dysfunction: (a) ALT and AST value were more than ten times higher than the upper limit of normal value; (b) serum creatinine value was more than 1.5 times higher than the upper limit of normal value; (c) total bilirubin was more than 2 times the upper limit of normal value; (3) patients with biliary obstruction; (4) pregnant women (urine or serum pregnancy test positive) or lactating women; (5) other factors considered unsuitable by the researchers for this trial, or the situation that may increase the risk of subjects or interfere with the clinical trial.

Adult male C57BL/6 mice were purchased from Shanghai Jiesijie experimental animal Co., Ltd. (Shanghai, China). All mice were 6–8 weeks of age and fed under the following conditions: (1) 20–26 °C; (2) 40–70% relative humidity; (3) 12:12-h light-dark cycle; (4) independent ventilated cages (IVC). Handling of animals was conducted in accordance with the Guide for the Care and Use of Laboratory Animals and was approved by the SPHCC Ethics Committee.

### Procedures

Laboratory parameters, chest CT scans, treatment, and outcome data were collected according to the patients’ medical records. The mild and severe cases of COVID-19 were distinguished by pulmonary lesions based on chest CT. The mild COVID-19 patients did not have pulmonary lesions, while the severe COVID-19 patients had pulmonary lesions including GGO and/or consolidation. We first detected the plasma hyaluronan levels of COVID-19 patients (*n* = 158) in SPHCC using Enzyme-linked Immunol sorbent assay (ELISA) as described previously.^8^ The blood samples of these patients were collected at the first fay when they were hospitalized. Meanwhile, 20 healthy subjects were recruited to evaluate their plasma hyaluronan levels as the control group. The 48.43 ng/ml of hyaluronan was sensitive to distinguish COVID-19 patients and health subjects via ROC analysis. Then, we divided the COVID-19 patients into two groups (HA ≥ 48.43 ng/mL; HA < 48.43 ng/mL) and confirmed whether there are significant differences between hyaluronan and other clinical parameters (lymphocytes, C-reactive protein, D-dimer, and fibrinogen). The lesion regions in the lungs of COVID-19 patients were quantified by artificial intelligence (AI) as the previous description.^26,36^ Briefly, we segmented the infection regions of lungs in COVID-19 patients based on chest CT scans and calculated the mass and volume of these infection regions.^37^ Furthermore, we analyzed the correlation between hyaluronan and these clinical parameters.

Adult male mice were used to assess the impact of hyaluronan on the lung lesions, such as GGO and consolidation. After anesthesia, the 200–400 kDa of hyaluronan dissolved in 1× PBS was intratracheal to mice (60 mg/kg), while the 1× PBS treatment was as the control group. Then, the formation of lung lesions in two groups was monitored via QuantumGX microCT on day 4.

Moreover, we evaluated whether hymecromone can reduce hyaluronan levels in HEK293T and HUVEC cells. Twenty-four hours later, HEK293T and HUVEC were treated with DMSO or hymecromone (200 μg/mL), cell culture mediums were collected to detect the hyaluronan levels using the Hyaluronan DuoSet ELISA (R&D Systems) according to the manufacturer’s descriptions. Meanwhile, we extracted the total RNA of HEK293T and HUVEC cells treated with DMSO or hymecromone. As previously described in our study,^8^ quantitative RT-PCR (RT-qPCR) was performed to evaluate whether hymecromone can decrease the expression of *HAS1*, *HAS2*, and *HAS3* (*HAS1/2/3*), which are the known hyaluronic acid synthases. The expression of *GAPDH* served as the normalized endogenous control. Relative mRNA expression was calculated via the 2^-ΔΔCt^ method (The primers are shown in Supplementary Table 2).

Finally, due to the limitation of hospitalized COVID-19 patients, 144 patients were recruited to conduct an open-label random trial to assess whether hymecromone could improve the clinical parameters of COVID-19 between Aug 1, 2020 and Mar 13, 2021. COVID-19 patients were divided into different groups based on the number at the end of the hospitalization number. The odd was as treatment group whereas the even was as the control group. Among these patients, 94 patients were oral hymecromone administration (2 tablets, 0.2 g/tablet, three times a day, before meals) combined with conventional treatment as the experimental group. The other 50 patients only underwent conventional treatment as the control group. Administration of hymecromone continued until the patients recovered from COVID-19. After they were recruited in this clinical trial, we collected their blood samples to evaluate the relevant parameters at the first day, fifth day, eleventh day, and the last day when they were in the hospital. Chest CT scans, lymphocyte counts, CRP, D-dimer, fibrinogen, and plasma hyaluronan were critical clinical indicators during COVID-19 patient treatment. The definition of lymphocytopenia, elevation of CRP, D-dimer, and fibrinogen satisfies the following criteria: (1) lymphocyte counts < 1.10 × 10^9^ /L; (2) CRP > 10.00 mg/mL; (3) D-dimer > 0.50 μg/mL; (4) fibrinogen > 4.00 g/L.

### Outcomes

Outcomes of COVID-19 patients were assessed based on the clinical indicators and chest CT images. The primary outcomes were the changes in lymphocyte counts, CRP, fibrinogen, and D-dimer in patients. The secondary outcome was the change in the patients’ chest CT results. We also monitored the suspected serious adverse reactions in accordance with regulatory requirements.

### Statistical analysis

The COVID-19 patients with pulmonary lesions were defined as severe while the others were considered mild. Twenty health subjects without any clinical symptoms were defined as the normal group. Samples (*n* = 158) were collected to compare the levels of hyaluronan in these three groups using the Mann–Whitney test. ROC analysis was used to identify the hyaluronan concentration for distinguishing normal groups and COVID-19 patients. Based on this analysis, COVID-19 patients were divided into two groups (HA ≥ 48.43 ng/mL; HA < 48.43 ng/mL). The significant differences between typical clinical indicators (such as lymphocytes, C-reactive protein, D-dimer, and fibrinogen) in these two groups were calculated by the Mann–Whitney test. Two-tailed Spearman’s correlation analysis was performed to evaluate the correlation between hyaluronan and these clinical indicators. Unpaired *t* test was used to confirm the significant differences between hyaluronan and *HAS1/2/3* expression among hymecromone-treated cells and DMSO-treated cells. Statistical analysis was performed via GraphPad Prism.

In addition, 144 COVID-19 patients were recruited to assess the effect of hymecromone for COVID-19. We further analyzed the effect of hymecromone using these patients with abnormal clinical parameters, including lymphocytes, C-reactive protein, D-dimer, and fibrinogen. For the primary outcomes, we calculated the fold changes of these clinical parameters per day in the hymecromone-treated group and control group and compared the clinical significances using the Mann–Whitney test. For the secondary outcome of change in chest CT results, we compared the hymecromone-treatment group and the control group, including the patients with GGO. According to CT quantitative analysis, we evaluated the change of pulmonary lesions, including improvement and exacerbation in COVID-19 patients classified by different hospitalization days (X < 14 day; 14 day ≤ X < 28 day; 28 day ≤ X < 35 day; X represents the hospitalization days). We calculated the percentage of improvement and exacerbation in different groups and analyzed the significance between the hymecromone-treated group and the control group using Fisher’s exact tests. The *P* value less than 0.05 was statistically significant (*P < 0.05; **P < 0.01; ***P < 0.001; ****P < 0.0001; ns, not significant).

## Supplementary information

Supplementary_Materials

## Data Availability

The data presented in this study have been included in the article or Supplementary Material. Further requests could be made to the corresponding authors.

## References

[1] Baj, A. et al. Breakthrough infections of E484K-harboring SARS-CoV-2 delta variant, Lombardy, Italy. *Emerg. Infect. Dis.* **27**, 3180 (2021). 10.3201/eid2712.211792 PMC8632179 34499599

[2] Kabinger F (2021). Mechanism of molnupiravir-induced SARS-CoV-2 mutagenesis. Nat. Struct. Mol. Biol..

[3] Owen DR (2021). An oral SARS-CoV-2 M(pro) inhibitor clinical candidate for the treatment of COVID-19. Science.

[4] Hurt, A. C. & Wheatley, A. K. Neutralizing antibody therapeutics for COVID-19. *Viruses* **13**, 628 (2021). 10.3390/v13040628 PMC8067572 33916927

[5] Xiang, R. et al. Recent advances in developing small-molecule inhibitors against SARS-CoV-2. *Acta Pharm. Sin. B* (2021). 10.1016/j.apsb.2021.06.016 PMC8260826 34249607

[6] Cui W, Yang K, Yang H (2020). Recent progress in the drug development targeting SARS-CoV-2 main protease as treatment for COVID-19. Front. Mol. Biosci..

[7] Du L, Yang Y, Zhang X, Li F (2022). Recent advances in nanotechnology-based COVID-19 vaccines and therapeutic antibodies. Nanoscale.

[8] Li W (2022). SARS-CoV-2 RNA elements share human sequence identity and upregulate hyaluronan via NamiRNA-enhancer network. eBioMedicine.

[9] Xu Z (2020). Pathological findings of COVID-19 associated with acute respiratory distress syndrome. Lancet Respiratory Med..

[10] Hällgren R, Samuelsson T, Laurent TC, Modig J (1989). Accumulation of hyaluronan (hyaluronic acid) in the lung in adult respiratory distress syndrome. Am. Rev. Respir. Dis..

[11] Hellman U (2020). Presence of hyaluronan in lung alveoli in severe Covid-19: an opening for new treatment options?. J. Biol. Chem..

[12] Liang J, Jiang D, Noble PW (2016). Hyaluronan as a therapeutic target in human diseases. Adv. Drug Deliv. Rev..

[13] Guan WJ (2020). Clinical characteristics of coronavirus disease 2019 in China. N. Engl. J. Med.

[14] Huang C (2020). Clinical features of patients infected with 2019 novel coronavirus in Wuhan, China. Lancet.

[15] Nagy N (2015). 4-methylumbelliferone treatment and hyaluronan inhibition as a therapeutic strategy in inflammation, autoimmunity, and cancer. Front. Immunol..

[16] Richardson S (2020). Presenting characteristics, comorbidities, and outcomes among 5700 patients hospitalized with COVID-19 in the New York City area. JAMA.

[17] Ruffell B, Johnson P (2008). Hyaluronan induces cell death in activated T cells through CD44. J. Immunol..

[18] Zhao W, Zhong Z, Xie X, Yu Q, Liu J (2020). Relation between chest CT findings and clinical conditions of coronavirus disease (COVID-19) pneumonia: a multicenter study. AJR Am. J. Roentgenol..

[19] Jacobs C (2014). Automatic detection of subsolid pulmonary nodules in thoracic computed tomography images. Med. Image Anal..

[20] Shi Y (2020). COVID-19 infection: the perspectives on immune responses. Cell Death Differ..

[21] Kultti A (2009). 4-Methylumbelliferone inhibits hyaluronan synthesis by depletion of cellular UDP-glucuronic acid and downregulation of hyaluronan synthase 2 and 3. Exp. Cell Res..

[22] Andonegui-Elguera, S. et al. Molecular alterations prompted by SARS-CoV-2 infection: induction of hyaluronan, glycosaminoglycan and mucopolysaccharide metabolism. *Arch. Med. Res.* **51**, 645–653 (2020). 10.1016/j.arcmed.2020.06.011 PMC7301110 32611485

[23] Ding M, Zhang Q, Li Q, Wu T, Huang YZ (2020). Correlation analysis of the severity and clinical prognosis of 32 cases of patients with COVID-19. Respir. Med..

[24] Esposito AJ, Bhatraju PK, Stapleton RD, Wurfel MM, Mikacenic C (2017). Hyaluronic acid is associated with organ dysfunction in acute respiratory distress syndrome. Crit. Care.

[25] Ponti G, Maccaferri M, Ruini C, Tomasi A, Ozben T (2020). Biomarkers associated with COVID-19 disease progression. Crit. Rev. Clin. Lab. Sci..

[26] Shi W (2021). A deep learning-based quantitative computed tomography model for predicting the severity of COVID-19: a retrospective study of 196 patients. Ann. Transl. Med.

[27] Yang, S. et al. Clinical and CT features of early stage patients with COVID-19: a retrospective analysis of imported cases in Shanghai, China. *Eur. Respir. J.* **55**, 2000407 (2020). 10.1183/13993003.00407-2020 PMC7098483 32217649

[28] Zhang W (2020). The characteristics and predictive role of lymphocyte subsets in COVID-19 patients. Int. J. Infect. Dis..

[29] Wiersinga, W. J., Rhodes, A., Cheng, A. C., Peacock, S. J. & Prescott, H. C. Pathophysiology, transmission, diagnosis, and treatment of coronavirus disease 2019 (COVID-19): a review. *J. Am. Med. Assoc.* **324**, 782–793 (2020). 10.1001/jama.2020.12839 32648899

[30] Group, R. C. et al. Dexamethasone in hospitalized patients with Covid-19. *N. Engl. J. Med.* **384***,* 693–704 (2021). 10.1056/NEJMoa2021436 PMC7383595 32678530

[31] Tomazini BM (2020). Effect of dexamethasone on days alive and ventilator-free in patients with moderate or severe acute respiratory distress syndrome and COVID-19: The CoDEX Randomized Clinical Trial. J. Am. Med. Assoc..

[32] Luo P (2020). Metformin treatment was associated with decreased mortality in COVID-19 patients with diabetes in a retrospective analysis. Am. J. Trop. Med Hyg..

[33] Gebhardt C (2010). Dermal hyaluronan is rapidly reduced by topical treatment with glucocorticoids. J. Investig. Dermatol..

[34] Sainio A (2020). Metformin decreases hyaluronan synthesis by vascular smooth muscle cells. J. Investig. Med..

[35] Li Q, Wang L, Wang B, Lu H (2021). The COVID-19-designated hospitals in China: preparing for public health emergencies. Emerg. Microbes Infect..

[36] Shan F (2021). Abnormal lung quantification in chest CT images of COVID-19 patients with deep learning and its application to severity prediction. Med Phys..

[37] Song YS (2014). Volume and mass doubling times of persistent pulmonary subsolid nodules detected in patients without known malignancy. Radiology.

