# Supplementary material for: Hymecromone: a clinical prescription hyaluronan inhibitor for efficiently blocking COVID-19 progression
Source: Signal Transduct Target Ther. 2022 Mar 18;7:91. doi: 10.1038/s41392-022-00952-w (PMC8931182; doi:10.1038/s41392-022-00952-w)
Supplement: Supplementary file 1 — Supplementary_Materials [file 41392_2022_952_MOESM1_ESM.docx]

Supplementary Materials for

Hymecromone: a clinical prescription hyaluronan inhibitor for efficiently blocking COVID-19 progression

Shuai Yang^1^, Yun Ling^2^, Fang Zhao^3^, Wei Li^1^, Zhigang Song^2^, Lu Wang^4^, Qiuting Li^1^, Mengxing Liu^1^, Ying Tong^1^, Lu Chen^1^, Daoping Ru^1^, Tongsheng Zhang^1^, Kaicheng Zhou^1^, Baolong Zhang^1^, Peng Xu^1^, Zhicong Yang^1^, Wenxuan Li^1^, Yuanlin Song^4^, Jianqing Xu^2^, Tongyu Zhu^4^, Fei Shan^3^, Wenqiang Yu^1^, and Hongzhou Lu^2,5^

Correspondence to: Prof. F Shan ([shanfei@shphc.org.cn](mailto:shanfei@shphc.org.cn)), Prof. W.Q. Yu ([wenqiangyu@fudan.edu.cn](mailto:wenqiangyu@fudan.edu.cn)), and Prof. H.Z. Lu (luhongzhou@fudan.edu.cn).

**This PDF file includes:**

Figures S1 to S6

Tables S1 to S2

Supplementary Figures


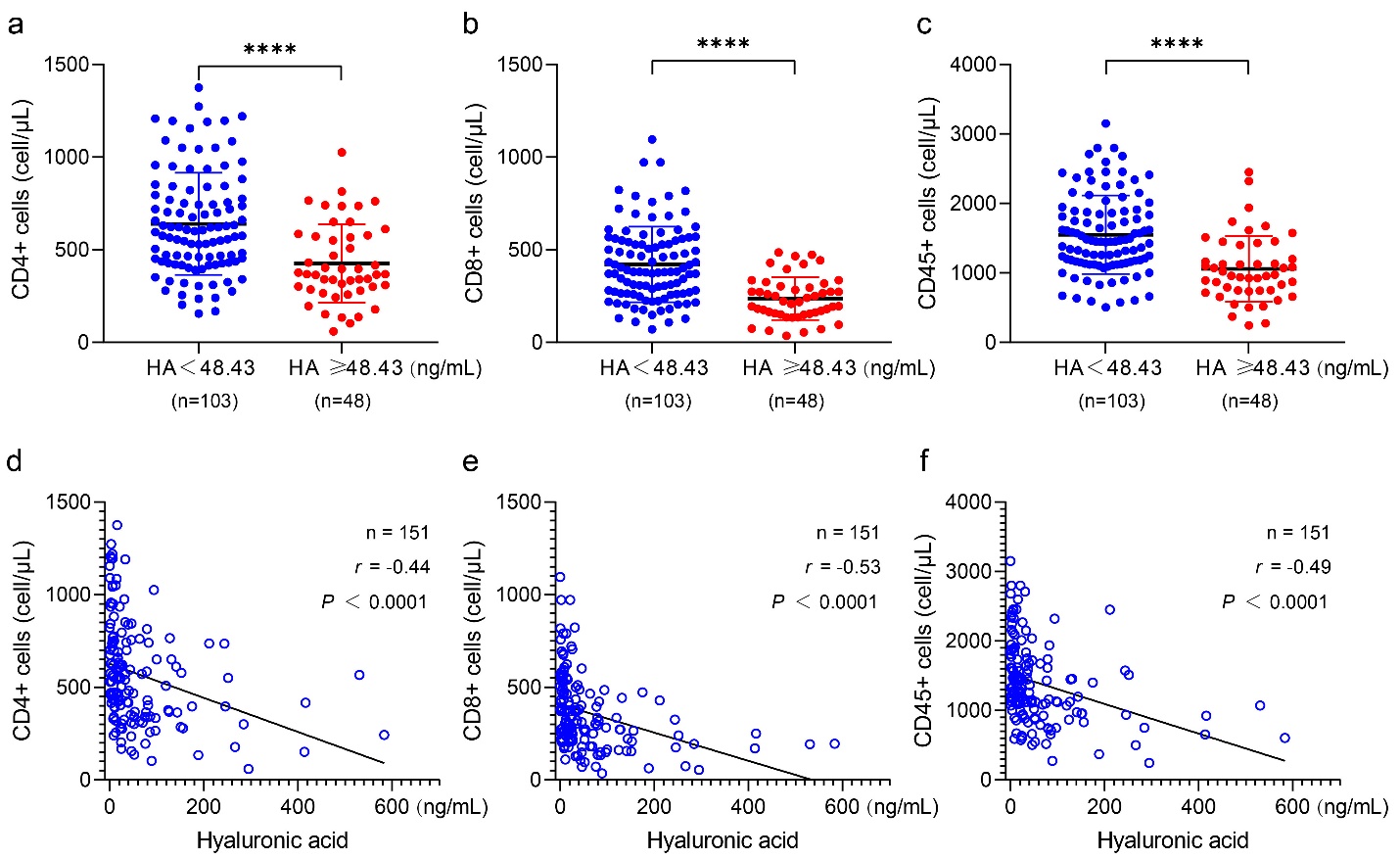


Figure. S1.

Correlation between hyaluronan and subtype of lymphocytes cell counts in COVID-19 patients. **a-c** The scatter plots showing CD4^+^ cell counts (**a**), CD8^+^ cell counts (**b**), and CD45^+^ cell counts (**c**) of COVID-19 patients classified by the threshold of 48.43 ng/mL of hyaluronan. Data was expressed by Mean ± SD. The significant difference was confirmed by Mann–Whitney test. *, *P* < 0.05; **, *P* < 0.01; ***, *P* < 0.001; ****, *P* < 0.0001; ns, not significant. **d-f** Counts of CD4^+^ cells (**d**), CD8^+^ cells (**e**), and CD45^+^ cells (**f**) upon admission plotted against hyaluronic acid, calculated by two-tailed Spearman’s correlation analysis.


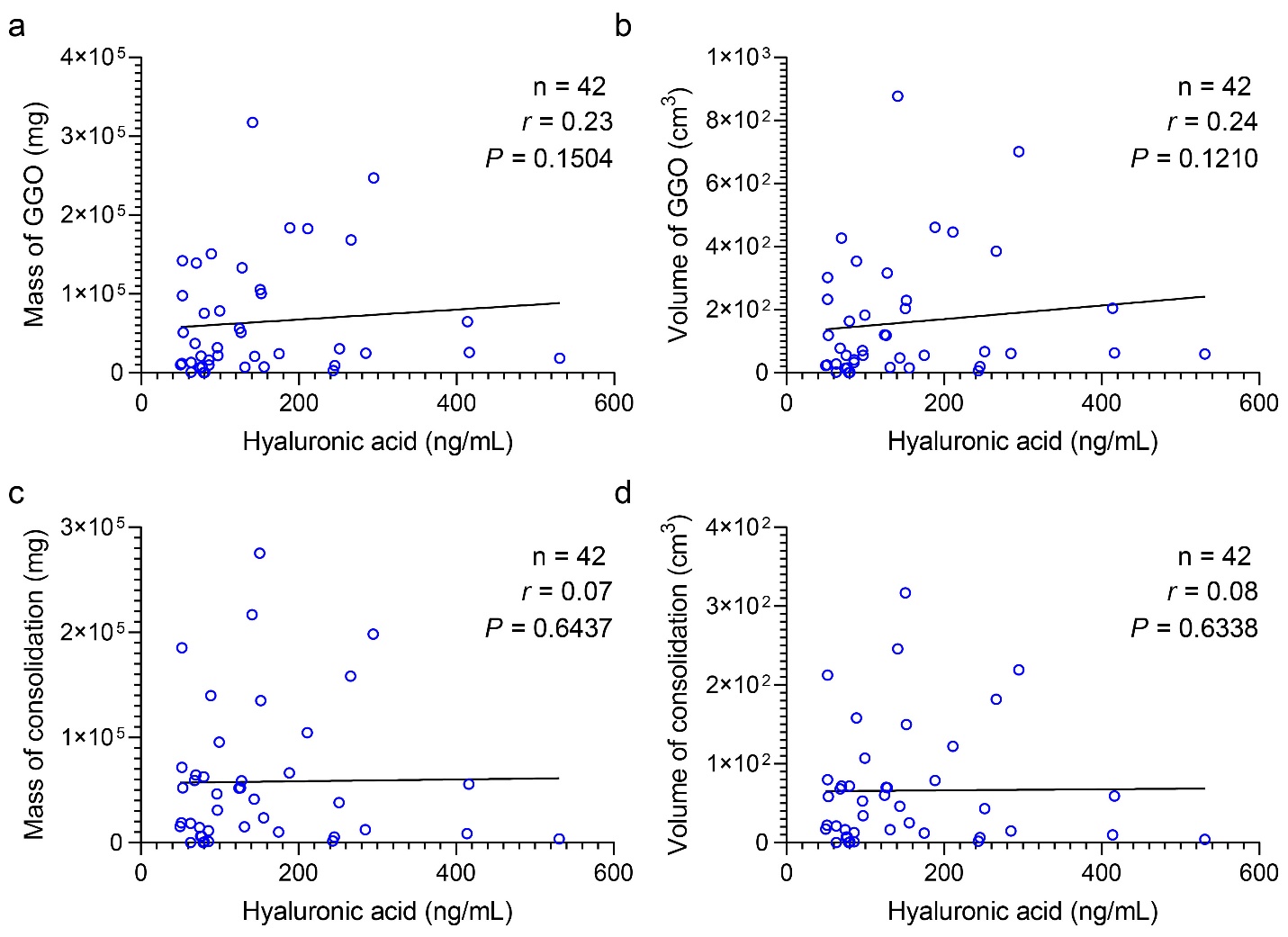


Figure. S2.

Correlation between hyaluronan and pulmonary lesions in COVID-19 patients with high level of hyaluronan (HA ≥ 48.43 ng/mL). **a-b** Scatter plot showing relation on the mass (**a**) and volume (**b**) of GGO against hyaluronic acid. **c-d** Scatter plot showing relation on the mass (**c**) and volume (**d**) of the consolidation region against hyaluronic acid. Mass and volume of pulmonary lesions were calculated via the automatic lung segmentation technology of AI based on CT images of severe COVID-19 patients. GGO was defined a range from -750 HU to -300 HU, and the consolidation region was defined from -300 HU to 50 HU. Two-tailed Spearman’s correlation analysis was performed to identify the relation of pulmonary lesions against hyaluronic acid.


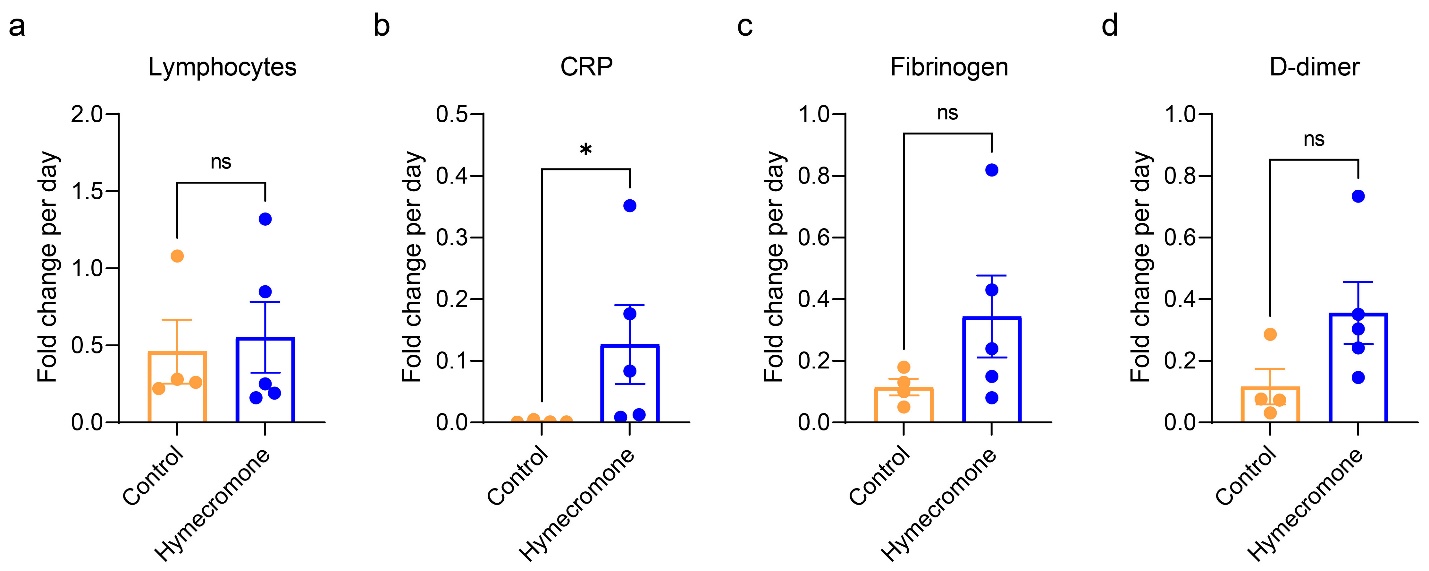


Figure. S3.

Hymecromone helps to decrease the CRP in COVID-19 patients. Changes in lymphocytes (**a**), CRP (**b**), fibrinogen (**c**), and D-dimer (**d**) were calculated as the fold change of diverse clinical indicators per day in patients with CRP elevation. Data was presented by Mean ± SEM. The significant difference was confirmed by the Mann–Whitney test. *, *P* < 0.05; **, *P* < 0.01; ***, *P* < 0.001; ****, *P* < 0.0001; ns, not significant.


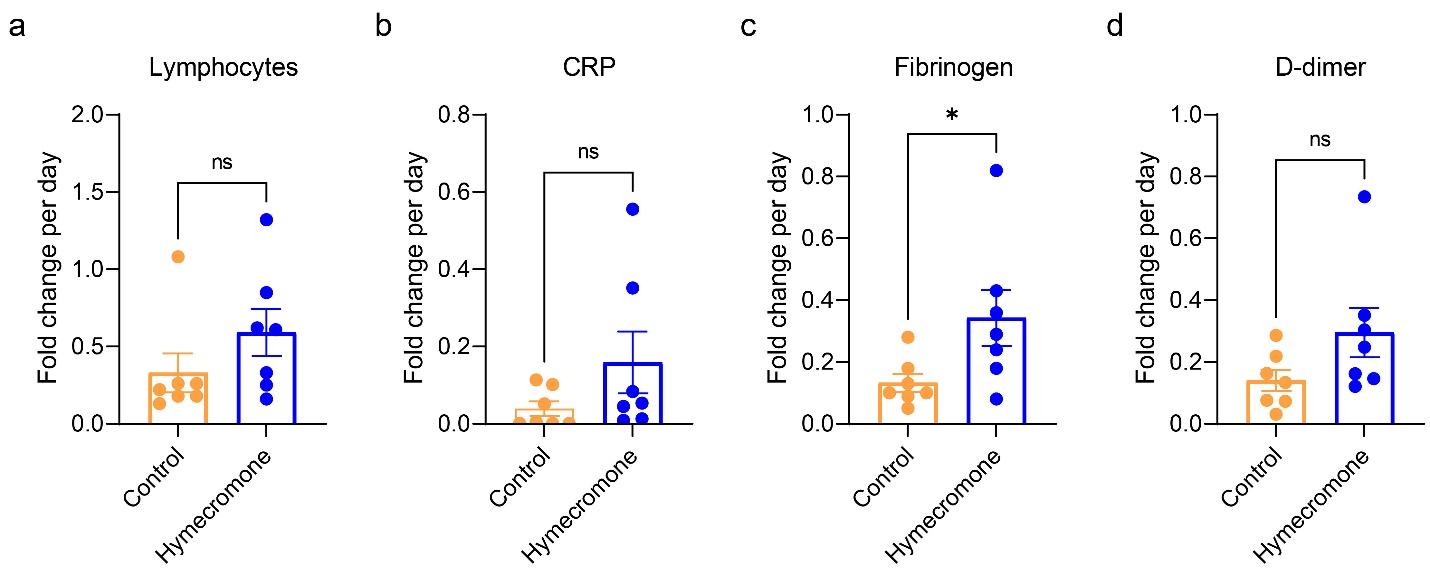


Figure. S4.

Hymecromone helps to decrease fibrinogen in COVID-19 patients. Changes in lymphocytes (**a**), CRP (**b**), fibrinogen (**c**), and D-dimer (**d**) were calculated as the fold change of diverse clinical indicators per day in patients with fibrinogen elevation. Data was presented by Mean ± SEM. The significant difference was confirmed by the Mann–Whitney test. *, *P* < 0.05; **, *P* < 0.01; ***, *P* < 0.001; ****, *P* < 0.0001; ns, not significant.


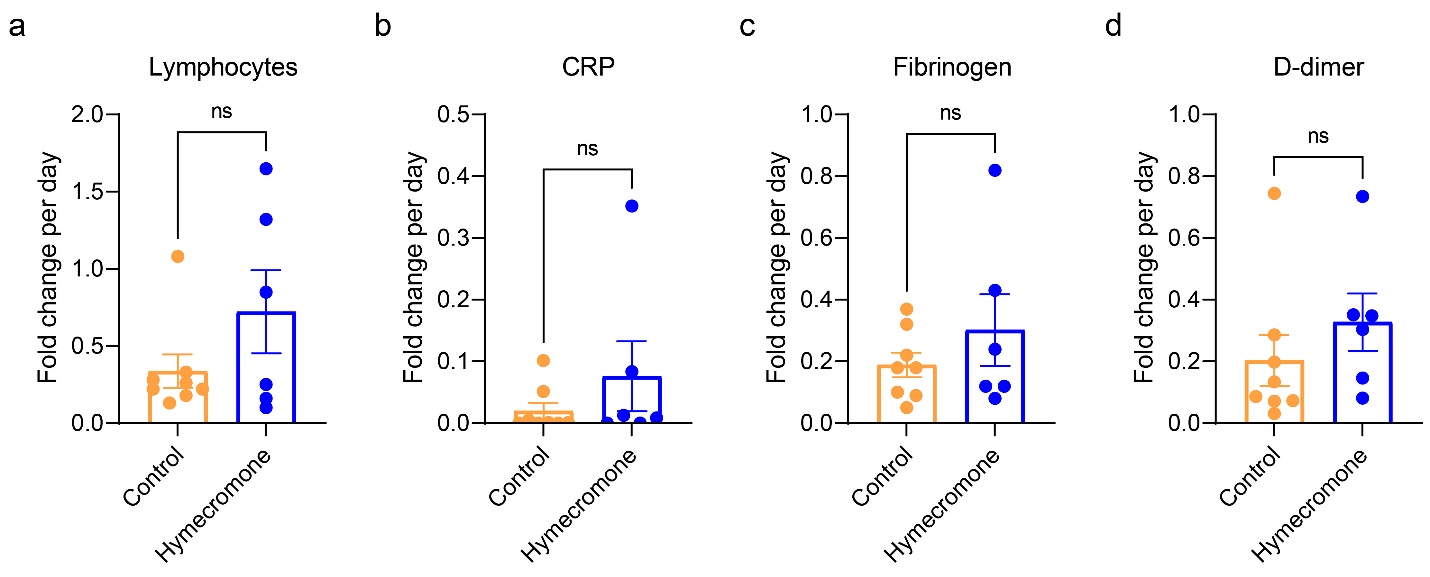


Figure. S5.

The effect of hymecromone on the elevation of D-dimer in COVID-19 patients. Changes in lymphocytes (**a**), CRP (**b**), fibrinogen (**c**), and D-dimer (**d**) were calculated as the fold change of diverse clinical indicators per day in patients with D-dimer elevation. Data was presented by Mean ± SEM. The significant difference was confirmed by the Mann–Whitney test. *, *P* < 0.05; **, *P* < 0.01; ***, *P* < 0.001; ****, *P* < 0.0001; ns, not significant.


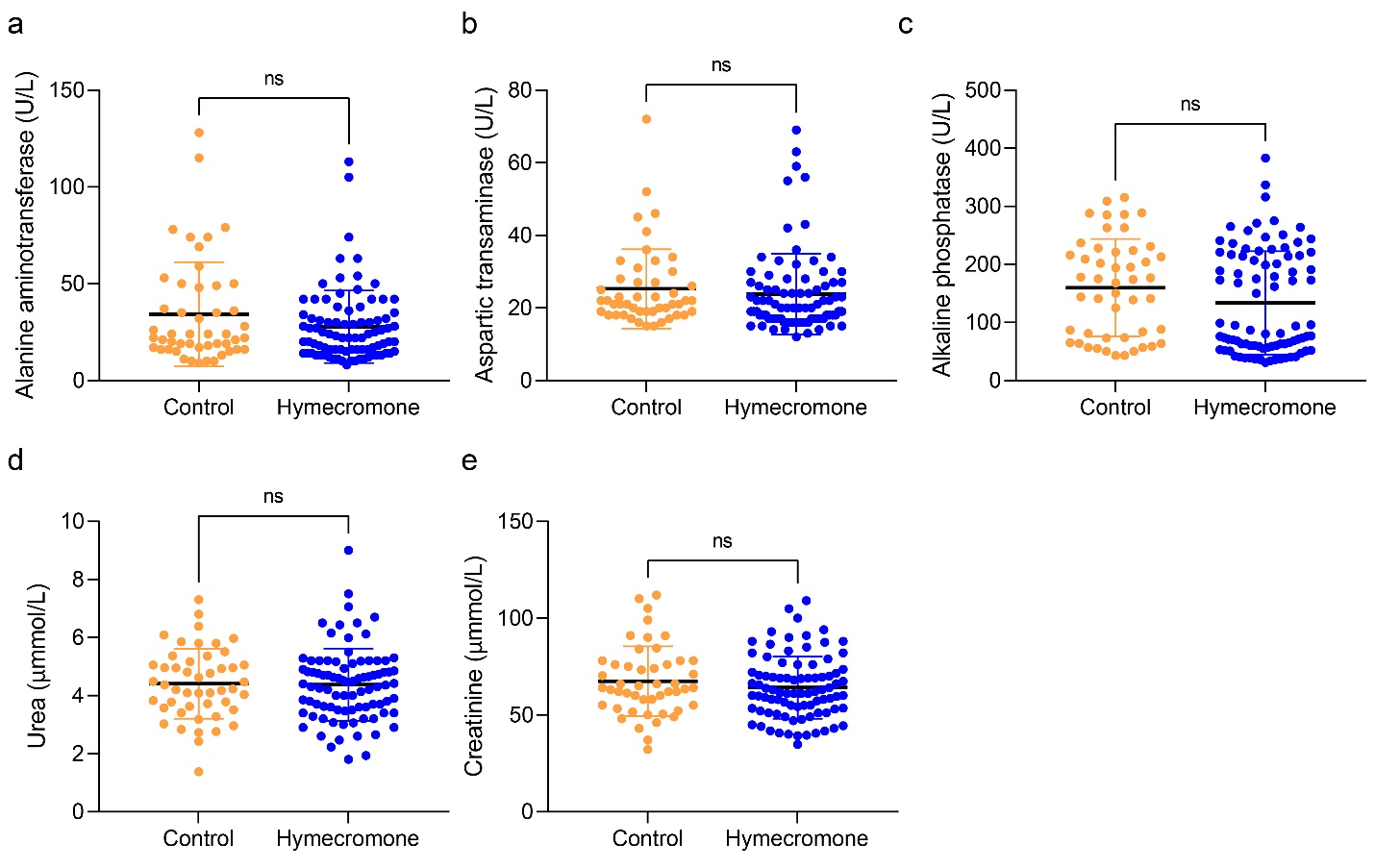


Figure. S6.

Comparison of the function of liver and kidney in control and hymecromone-treated groups. **a-c** Alanine aminotransferase (ALT) (**a**), aspartic transaminase (AST) (**b**), and alkaline phosphatase (ALP) (**c**) were used to assess the function of liver. **d-e** Urea (**d**) and creatinine (**e**) were used to assess the function of kidney. Data was presented by Mean ± SD. The significant difference was confirmed by the Mann–Whitney test. *, *P* < 0.05; **, *P* < 0.01; ***, *P* < 0.001; ****, *P* < 0.0001; ns, not significant.

Supplementary Tables

Table S1.

Comparison of the age, gender, and end-point events in control and hymecromone treated groups.

|  | | Control group | Experimental group | *P* value |
| --- | --- | --- | --- | --- |
| Age, Median (range) | 35 (21-69) | 41.5 (21-77) | 0.1992^†^ |  |
| Female | 14 (28.00%) | 31 (32.98%) | 0.5763^‡^ |  |
| Male | 36 (72.00%) | 63 (67.02%) |  |  |
| Lymphopenia | 5 (10.00%) | 8 (8.51%) | 0.7672^‡^ |  |
| CRP elevation | 4 (8.00%) | 5 (5.32%) | 0.7193^‡^ |  |
| Fibrinogen elevation | 7 (14.00%) | 7 (7.45%) | 0.2425^‡^ |  |
| D-dimer elevation | 8 (16.00%) | 6 (6.38%) | 0.0792^‡^ |  |

^†^ *P* value was calculated by Mann-Whitney test using GraphPad Prism. ^‡^*P* value was calculated by Fisher's exact test using GraphPad Prism.

Table S2.

The primer sequences for RT-qPCR.

| **Primers** | **Sequence (5'-3')** |
| --- | --- |
| GAPDH-F | CTGGGCTACACTGAGCACC |
| GAPDH-R | AAGTGGTCGTTGAGGGCAATG |
| HAS1-F | CGCTCGGAGATTCGGTGGACTA |
| HAS1-R | CCAGTATCGCAGGCTGCTTAGG |
| HAS2-F | GCTCGCAACACGTAACGCAAT |
| HAS2-R | CCAGTGCTCTGAAGGCTGTGTA |
| HAS3-F | AGGTGGTCATGGTGGTGGATGG |
| HAS3-R | CTCGCCTGCCTCATGGAAGTTG |
